# Neuroprotective effect of quercetin and *Zingiber officinale* on sodium arsenate‐induced neurotoxicity in rats

**DOI:** 10.1002/fsn3.3278

**Published:** 2023-02-28

**Authors:** Rasia Yousuf, Pawan Kumar Verma, Priyanka Sharma, Shilpa Sood, Muhammad A. Bhatti, Zuhaib F. Bhat

**Affiliations:** ^1^ Division of Veterinary Pharmacology and Toxicology, Faculty of Veterinary Science and Animal Husbandry SKUAST‐J Jammu India; ^2^ Division of Veterinary Pathology, Faculty of Veterinary Science and Animal Husbandry SKUAST‐J Jammu India; ^3^ Faculty of Landscape and Society International Environment and Development Studies, Noragric Norwegian University of Life Sciences (NMBU) Public university, Ås Norway; ^4^ Division of Livestock Products Technology SKUAST‐J Jammu India

**Keywords:** antioxidant status, arsenic, neurotoxicity, quercetin, Wistar rats, *Zingiber officinale*

## Abstract

The study was aimed at determining the ameliorative potential of quercetin and *Zingiber officinale* (ZO) against sodium arsenate‐induced neurotoxicity in male Wistar rats. Thirty adult animals were randomly allocated to five groups (*n* = 6). Group I served as control, groups II and IV were treated with ZO [300 mg/kg, PO (per os)/day], and group V animals were administered quercetin (50 mg/kg, PO/day) for 18 days. Groups III, IV, and V were injected with sodium arsenate (20 mg/kg, intraperitoneally/day) for 4 days starting from day 15. The administration of sodium arsenate resulted in a significant decrease in total antioxidant status, total thiols, superoxide dismutase, catalase, glutathione peroxidase, glutathione reductase, and aryl esterase in brain tissue of the animals compared with the control group. In addition, a significant increase was observed in malondialdehyde, advanced oxidation protein product and plasma nitric oxide levels, indicating oxidative stress‐mediated neuronal damage. However, these arsenic‐induced alterations were significantly reversed by quercetin or ZO in the treatment groups, indicating their ameliorative potential. These positive effects were further confirmed by histopathological examination of brain tissue revealing the suppression of severe neuronal injury, spongiosis and gliosis in the samples pretreated with quercetin and ZO. Our results suggest that inclusion of ZO and quercetin‐rich foods in the diet can help in preventing the neurotoxic effects in areas with elevated levels of arsenic in food chain and ground water.

## Graphical abstract

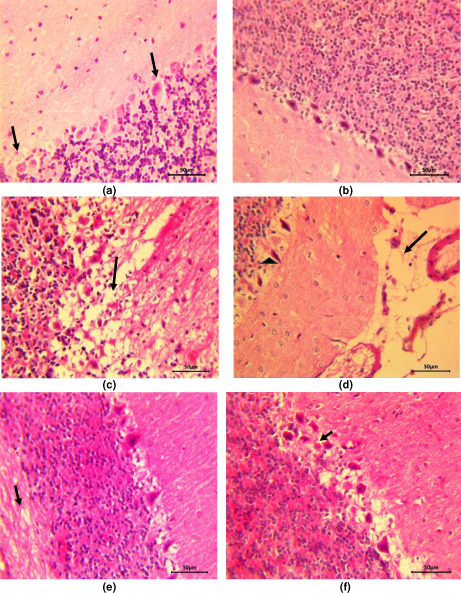

## 1 INTRODUCTION

Environmental contamination by heavy metals poses a serious global public health hazard, endangering millions of lives worldwide. Arsenic (As), a highly toxic heavy metal, is an ubiquitous pollutant due to its natural occurrence in earth's crust as well as its emission due to wide range of anthropogenic activities (Chandravanshi et al., 2019; Seif et al., 2021). Exposure to unacceptable amounts of arsenic is associated with increased risk of different types of ailments, including cancers, neurological disorders, loss of memory and depression (Coryell et al., 2019; Sodhi et al., 2019). The maximum limit set by the World Health Organization (WHO) and environmental protection agency (EPA) for arsenic in drinking water is 10 ppb, but levels up to 50 ppb are acceptable in some Asian countries like Bangladesh (Alam et al., 2018; Peruru et al., 2020; Singh et al., 2020). Arsenic crosses blood– brain barrier and gets deposited in different parts of the brain, such as cortex, cerebellum, hypothalamus, and hippocampus, resulting in a variety of neuropathological conditions (Peruru et al., 2020; Thakur et al., 2021). Data from previous experimental and clinical investigations suggest that arsenic intoxication disrupts neuronal architecture and significantly impairs neurophysiological processes which can predispose to cognitive dysfunctioning and dementia as seen in neurodegenerative disorders, such as Alzheimer's disease (Rahman et al., 2021).

Nitric oxide (NO) is a high reactive endogenous molecule that plays an imperative role in neurological disorders. Neuronal nitric oxide synthase (nNOS) induced NO in brain act as a neurotransmitter to regulate various physiological processes, such as cognitive function and synaptic plasticity. It also controls brain blood flow and promotes angiogenesis and maintains cellular redox potential, immunity, and neuronal survival (Levine et al., 2012; Luiking et al., 2010). However, excessive production of NO alone or in combination with other superoxide radicals leads to neurotoxicity and consequent neurodegeneration. NO causes post‐translational modifications in proteins by S‐nitrosylation of the thiols which regulate protein function physiologically. This process of protein nitrotyrosination is irreversible and results in the accumulation of the modified proteins which contribute to the progression of neurodegeneration in mammalian brain (Knott & Bossy‐Wetzel, 2009; Mishra & Flora, 2008). Mechanistic studies have revealed that oxidative insult due to excessive reactive oxygen and nitrogen species (ROS/RNS) generation and/or reduced radical scavenging capacity is a major contributor in the development of arsenic‐induced structural and functional neurological abnormalities (Li et al., 2021; Thakur et al., 2021). Arsenic toxicity impairs mitochondrial functioning and exacerbates electron leakage which together with high levels of intermediate arsenic metabolites and arsenic enhances lipid peroxidation (Li et al., 2021). Brain requires proportionately higher oxygen levels for its proper functioning as neurons derive energy through oxidative phosphorylation. The presence of a robust redox signaling and relative deficiency in antioxidant defense makes the brain more susceptible to oxidative insults (Cobley et al., 2018). Being rich in lipid content and its inability for cellular regeneration or repair increases vulnerability of brain to oxidative damage induced by exposure to toxicants such as arsenic (Garza‐Lombó et al., 2019).

Imminent prospect of unintended exposure to various neurotoxicants like arsenic prevailing in the polluted ecosystem are suspected to potentiate the development and severity of different neurodegenerative disorders. There is a growing interest among scientific community to explore newer prophylactic and ameliorative strategies to counter these toxicants‐induced neurological disorders. Many recent research studies have highlighted effective prevention and amelioration of arsenic‐induced neurotoxicity using different plant‐based interventions (Peruru et al., 2020; Susan et al., 2019; Yadav et al., 2011). The constituent phytochemicals in these herbal preparations displayed prophylactic antioxidant and anti‐inflammatory properties which were responsible for countering arsenic‐mediated toxicity. The quercetin is one of the most common phytochemicals that has potent protective properties against oxidative neurological damage. It is widely present in dry fruits and has potent antioxidant capabilities and may protect the brain tissue from oxidative stress associated disorders (Ishisaka et al., 2011; Mishra & Flora, 2008). The exact mechanism is unknown but several mechanisms have been attributed such as inhibition of nuclear factor‐kappa B (NF‐kappa B), suppression of over‐expression of the iNOS, activation and modulation of C‐reactive protein and cyclooxygenase‐2 (Zhang et al., 2009). The phytochemicals present in some plant extracts possess free radical scavenging capabilities and are also good at chelating extraneous ions and alter NO level in brain and therefore can prove beneficial in the neutralization and elimination of arsenic from the body. One such source is ginger (*Zingiber officinale* Roscoe, belong to family *Zingiberacae*), commonly utilized as a spice or supplement in beverages and food products. Ginger harbors exceptional remedial antioxidant capabilities and was shown to be more powerful than many other natural products in the management of ailments and toxicities including those due to arsenic exposure (Seif et al., 2021; Sharifi‐Rad et al., 2017). Phytochemically, *Z. officinale* (ZO) is very rich in flavonoids, alkaloids, tannins, and phenolic components, such as shogaols, zingerone, and gingerols. Therefore, the present study was designed to evaluate the neuroprotective potential of quercetin and ZO extract in sodium arsenate‐induced neurotoxic rats.

## 2 MATERIALS AND METHODS

### 2.1 Extract preparation

The rhizome part of the plant *Zingiber officinale* was collected from the local market and identified by the Taxonomists of University of Kashmir. After proper identification of plant rhizome, sufficient quantity of the sample was collected, cleaned, and dried in the laboratory. Dried parts were pre‐crushed and pulverized into fine powder using electric grinder. Powdered rhizome parts were subjected to hydro‐alcoholic extraction (1:1) using Soxhlet apparatus maintaining hot plate temperature between 65–70°C. The final drying was done in a rotatory evaporator (55–60°C, 15 rpm). The dried extract was stored in a glass container in a refrigerator until used.

### 2.2 Experimental design and animals

The experiments were performed using adult male Wistar rats purchased from the Indian Institute of Integrative Medicine (CSIR‐Lab), Jammu, India. The animals were acclimatized for 1 week on pelleted feed with access to ad lib potable water under standard conditions [25 ± 2°C temperature, 50 ± 15% relative humidity and normal photoperiod (12 h light and dark cycle)]. The pelleted feed provided to rats was supplied by Animal Feed Unit, Department of Animal Nutrition, CSKHPKV, Palampur, Himachal Pradesh, India. All the experimental animals were kept under observation during the entire period of study. The study was duly approved by Institutional Animal Ethics Committee (IAEC) of F.V.Sc. & A.H., SKUAST‐Jammu (3/IAEC/2020). The experimentation was conducted as per the standard regulatory guidelines. Thirty adult male Wistar rats, weighing between 180 and 200 g, were randomly allocated to five groups with 6 animals in each group (*n* = 6). Group I served as control, group II animals were given hydro‐alcoholic extract (1:1) of *Z. officinale* [300 mg/kg body weight (BW), per os (PO), for 18 days]. Animals of group III were treated with sodium arsenate [20 mg/kg, intraperitoneally (IP)] daily for 4 days and this treatment in group III coincided with the last 4 days of extract treatment of group II. Group IV and group V were treated with *Z. officinale* extract (300 mg/kg BW, PO) and quercetin (50 mg/kg BW, PO), respectively, daily for 18 days and both the groups were additionally exposed to sodium arsenate (20 mg/kg/day, IP) on the last 4 days of extract or quercetin daily administration. The rats were further monitored for 48 h before their final sacrifice. The dose of sodium arsenate used in our study to induce toxicity has been reported by the previous study (Rizwan et al., 2014). The body weights of all the animals were recorded prior to the treatments and after the completion of the treatments.

### 2.3 Collection of blood and tissues

Prior to sacrifice on day 20th of experimentation, blood samples were collected from diethyl ether anesthetized rats directly from heart in heparin‐containing tubes. Animals were sacrificed by cervical dislocation and brain tissue was removed and a portion of it was collected in 10 mL ice‐cold 0.5 M phosphate buffer (pH ‐7.4) for antioxidant studies and a part of cerebrum and cerebellum were collected in formal saline solution (10%) for histopathological studies. Tissue homogenate was prepared by homogenizing the brain tissue at 123 *g* for 5–7 min at room temperature.

### 2.4 Antioxidant biomarkers in brain tissue

The activities of antioxidant biomarkers and cellular damage indicators were estimated in nervous tissue of different groups of rats. The activity of arylesterase (AE) was measured by using phenyl acetate as a substrate and was expressed in unit per ml (U/ml), where one unit corresponds to μmol phenol formed per minute (Burlina et al., 1977). Total antioxidant status (TAS) and total thiols (TTH) levels were estimated using well‐known standard methods (Mochnik et al., 1994; Re et al., 1999). The enzymatic parameters viz., catalase (Aebi, 1974) and glutathione peroxidase (GPx) were determined following standard methods described by Hafeman et al. (1974). The activities of superoxide dismutase (SOD) and glutathione reductase (GR) were determined as per the method described elsewhere (Carlberg & Mannervik, 1985; Marklund & Marklund, 1974). The malondialdehyde (MDA) and advance oxidation protein product (AOPP) levels in brain tissue were determined using methods described by Shafiq‐Ur‐Rehman (1984) and Witko‐Sarsat et al. (1996). Levels of nitric oxide (NO) were measured in plasma of all the groups by spectrophotometric method using copper–cadmium alloy (Sastry et al., 2002). The principle of the assay is the reduction of nitrate to nitrite by copper–cadmium alloy, followed by color development on addition of Griess reagent (sulfanilamide and N‐naphthyl ethylenediamine) in acidic medium which is measured spectrophotometrically at 545 nm.

### 2.5 Histopathology

Samples from cerebrum and cerebellum from all the rats in different groups were collected in formalin. The formalin fixed brain tissues were dehydrated, cleared, paraffin embedded, sectioned, and stained with hematoxylin and eosin. The histological preparations were then observed under light microscope for the presence of any histomorphological changes.

### 2.6 Statistical analysis

Standard statistical procedures were followed and the data collected during the experiment was subjected to analysis of variance (ANOVA) using statistical software SPSS and the significance were tested using Duncan Multiple Range Test. The significance was assayed at 5% (*p* < .05) level.

## 3 RESULTS

### 3.1 Alterations in body weight and plasma nitric oxide (NO) level

**FIGURE 1:**
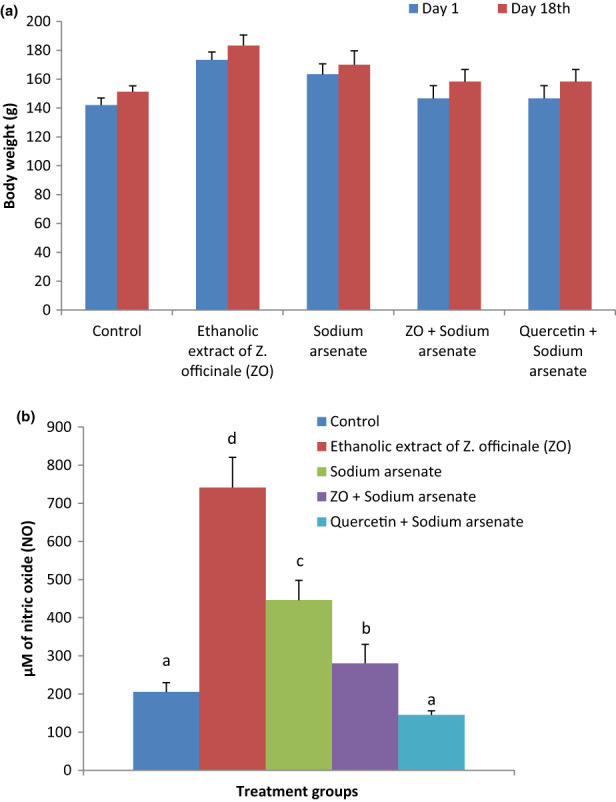
Changes in the body weight of animals on day 1 and day 18 of different treated groups (a) and plasma level of nitric oxide (NO) in Wistar rats following treatment of *Z. officinale* and sodium arsenate alone and along with *Z. officinale* and quercetin (b).

Alterations in body weight of rats following treatments are presented in Figure 1a. No significant effect of the treatments was observed on the body weight of the animals. Treatment with ZO and sodium arsenate did not alter body weight of rats significantly compared with body weight of corresponding animals before the start of experiment. The percent change in body weight between days 1 and 18 was lowest (4.08%) in sodium arsenate alone administered rats and highest in rats treated with ZO along with arsenic (7.95%). Alterations in NO levels in plasma of rats are presented in Figure 1b. A significant (*p* < .05) increase in mean NO level in plasma was observed in sodium arsenate (117.4%) treated group as compared with the control. Treatment with ZO followed by sodium arsenate exposure resulted in a significant lowering of NO levels than the corresponding levels in sodium arsenate alone exposed group. Decline in plasma NO level was also observed in animals treated with quercetin prior to sodium arsenate treatment but did not differ significantly from the respective levels in control rats.

### 3.2 Brain antioxidant biomarkers

Alterations in brain antioxidant biomarkers, thiols homeostasis, and tissue damage markers in different groups are presented in Tables 1, 2. Significant (*p* < .05) decline in mean TAS level (71.7%) was observed in sodium arsenate treated rats whereas animals treated with ZO showed a non‐significant (*p* > .05) increase (26.1%) compared with control group. Pretreatment with ZO followed by sodium arsenate exposure significantly increased (*p* < .05) brain TAS levels in comparison with the respective levels in the sodium arsenate only exposed group; however, the TAS values after co‐treatment with ZO and arsenic were still significantly lower (57.2%) than the control values. Similar trend was noted in arsenate administered rats pretreated with quercetin, wherein the TAS levels showed a significant enhancement in TAS levels compared with arsenic alone treated group. These values were significantly higher than the corresponding levels in group treated with sodium arsenate and ZO. A significant (*p* < .05) reduction in activities of AE (51.5%), CAT (27.2%), and SOD (21.9%) were observed in brain tissue in sodium arsenate‐treated group. In comparison, ZO pretreatment in sodium arsenate exposed rats resulted in a significant improvement in the levels of these enzymes (*p* < .05); however, these values remained significantly lower than the respective control values. In a similar fashion, group administered with quercetin and sodium arsenate exhibited a significant (*p* < .05) augmentation in AE and SOD activities but no significant difference in CAT activities as compared with the corresponding activities in arsenate alone treated rats. Strikingly, pretreatment with ZO caused a significantly higher restoration of AE and SOD activities in arsenate injected rats than the pretreatment with quercetin whereas no significant difference was seen in the extent of improvement in SOD activities between the two co‐treated groups.

**TABLE 1:** Protective effect of *Z. officinale* extract and quercetin on total antioxidant status, total thiols, and cellular damage indicator against sodium arsenate‐induced neurotoxicity in Wistar rats.

Treatment groups	Antioxidant biomarkers			
	TAS (mM)	TTH (μM)	MDA (nmoles MDA produced / g of tissue / hr)	AOPP (μM of chloramine T)
Control	20.70^d^ ± 0.532	2.04^b^ ± 0.061	50.14^a^ ± 1.05	1.31^bc^ ± 0.008
*Z. officinale*	26.10^d^ ± 0.222	2.15^b^ ± 0.080	50.82^a^ ± 1.37	1.26^ab^ ± 0.006
Sodium arsenate	5.86^a^ ± 0.273	1.37^a^ ± 0.017	169.13^d^ ± 2.45	1.41^d^ ± 0.013
*Z. officinale* + Sodium arsenate	8.87^b^ ± 0.523	1.47^a^ ± 0.010	148.89^c^ ± 2.25	1.30^bc^ ± 0.015
Quercetin + Sodium arsenate	17.41^c^ ± 0.597	1.47^a^ ± 0.015	66.06^b^ ± 2.07	1.37^d^ ± 0.022
*p*‐Value	a, b, c, d = 1.00	a = .141, b = .121	a = .805, b, c, d = 1	a, b = .056, c = .857, d = 1

*Note*: Values given are mean ± SE (*n* = 6). Mean values in a column having different superscript (a, b, c) differ significantly at 5% (*p* < .05) level of significance.

**TABLE 2:** Protective effect of *Z. officinale* extract and quercetin on antioxidant biomarkers against sodium arsenate‐induced neurotoxicity in Wistar rats.

Treatment groups	Antioxidant biomarkers				
	SOD (unit/ g of tissue)	CAT (μmol H_2_O_2_ decomposed/ min/ g of tissue)	GPx (unit/ g of tissue)	GR (nmol of NADPH/min)	AE (U/ml)
Control	1052.49^c^ ± 11.94	3398.89^d^ ± 94.63	237.04^b^ ± 4.24	33.20^d^ ± 0.69	2.72 ^e^ ± 0.04
*Z. officinale*	1062.64^d^ ± 7.03	3365.39^d^ ± 22.97	250.31^c^ ± 1.79	31.18^d^ ± 1.01	2.62^d^ ± 0.03
Sodium arsenate	821.73^a^ ± 10.96	2473.14^b^ ± 91.20	139.29^a^ ± 1.87	13.19^a^ ± 0.60	1.32^a^ ± 0.01
*Z. officinale* + Sodium arsenate	913.5^b^ ± 6.35	3134.09^c^ ± 54.71	235.16^b^ ± 1.34	17.23^b^ ± 0.54	2.07^c^ ± 0.04
Quercetin + Sodium arsenate	879.78^b^ ± 9.26	2794.41^b^ ± 44.62	145.18^a^ ± 2.87	24.36^c^ ± 0.63	1.71^b^ ± 0.01
*p*‐Value	a = 1, b = .059, c = .0556	a, b, c = 1, d = .729	a = .127, b = .620, c = 1	a, b, c = 1, d = .056	a, b, c, d = 1

*Note*: Values given are mean ± SE (*n* = 6). Mean values in a column having different superscript (a, b, c) differ significantly at 5% (*p* < .05) level of significance.

### 3.3 Thiols homeostasis of brain tissue

The TTH levels registered a significant (*p* < 0.05) decline (33.2%) in brain tissue following treatment with sodium arsenate as compared with corresponding values of control. Neither of the pretreatments (ZO or quercetin) could significantly alter the decline in TTH levels after arsenic intoxication. Repeated oral administration of ZO alone led to a significant increase (5.6%) in GPx activities whereas no change was seen in the GR activity in this group as compared with the corresponding control values. Significant (*p* < .05) decline in GPx (41.2%) and GR (60.3%) activities in brain were observed in sodium arsenate‐treated group as compared with the respective control levels. In rats pretreated with ZO and subsequently injected with sodium arsenate, GR activities remained significantly decreased as compared with the control levels but were significantly higher as compared with the levels in rats given arsenate treatment alone. Similarly, GPx activities showed a significant increase in rats pretreated with ZO followed by arsenic as compared with arsenic alone treated rats and these levels did not show any significant difference as compared with the control GPx activity. In contrast, arsenate toxicity in quercetin pretreated group did not cause any significant alterations in GPx activities as compared with the corresponding levels in arsenate only treated animals. The significant improvements in GPx and GR activities in ZO pretreated groups were significantly higher and lower, respectively, than quercetin pretreated groups.

### 3.4 Brain tissue damage indicators

During oxidative damage, membrane lipids, and proteins are oxidized to their respective end products, that is, MDA and AOPP, respectively. In the present study, a significant (*p* < .05) increase in the mean MDA levels (133.3%) was observed in brain tissue in sodium arsenate‐treated group as compared with the control group. The ZO pretreated rats subjected to arsenate toxicity showed a significant decline (*p* < .05) in MDA levels as compared with the corresponding values in animals receiving only arsenate. Nonetheless, MDA levels in pretreated as well as intoxicated animals were significantly higher than the respective values in the control group. MDA contents in animals who were pretreated with quercetin before arsenate intoxication were significantly lower than the arsenate only treated group as well as the rats pretreated with ZO. Also, a significant (*p* < .05) increase in AOPP levels (8.5%) in brain tissue was observed in sodium arsenate‐treated group as compared with control. Pretreatment with ZO followed by sodium arsenate exposure caused a significant (*p* < .05) lowering of AOPP levels in brain compared with the sodium arsenate alone group whereas no significant difference was seen in AOPP values between ZO alone, pretreated ZO, and control groups. On the contrary, quercetin pretreatment failed to induce any significant reduction in AOPP levels.

### 3.5 Histological alterations in brain

**FIGURE 2:**
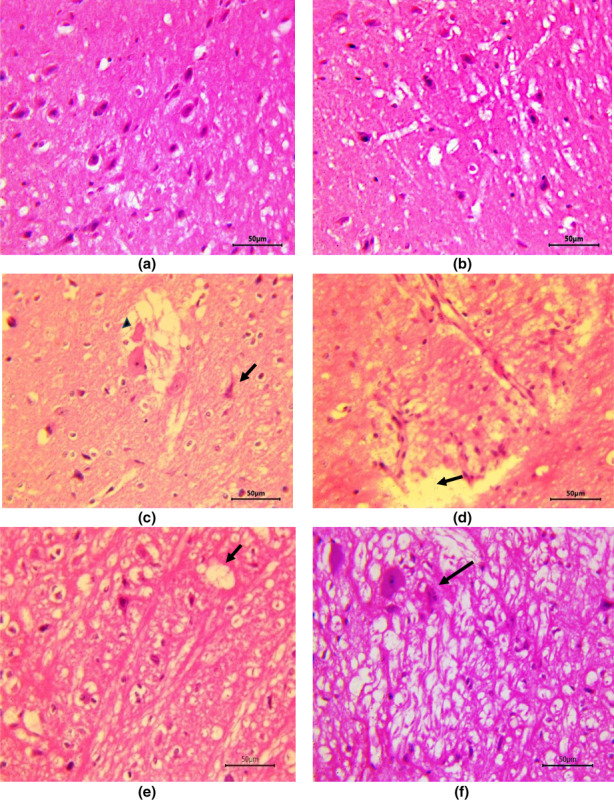
Histopathology of normal cerebral parenchyma was seen in control animals (a) and no pathological changes seen in cerebrum of the group II (b). Group III showed perineuronal edema (arrowhead), neuronal degeneration and necrosis (arrow) (c) and perivascular edema, endothelial hypertrophy and spongiosis (arrow) (d). Group IV rat cerebrum revealed mild spongiotic vacuolation (arrow) and neuronal degeneration (e), whereas in group V moderate spongiosis and neuronal degeneration (arrow) were observed (f).

Cerebrum in control group rats and group receiving ZO only did not reveal any deviation from normal histology (Figure 2a,b). Neurons in cerebral cortex of rats in these groups were normal with centrally placed nuclei and neuropil and myelinated tracts in white matter did not reveal any changes. In rats treated with sodium arsenate, multifocally, neurons were degenerated or necrotic appearing shrunken with deeply eosinophilic cytoplasm and pyknotic nuclei (Figure 2c). Many neurons had central chromatolysis and showed loss of nucleus (Figure 2c). Gliosis as evidenced by the presence of glial cells forming aggregates randomly throughout the parenchyma and neuronophagia, characterized by glial cells forming a rim around necrotic neuron, were also appreciated. Hemorrhages, congestion, perivascular edema along with endothelial hypertrophy as well as perineuronal edema were quite marked and neuropil was often severely spongiotic (Figure 2c,d). Meningeal congestion and edema along with lymphocytic infiltration were very prominent. The ZO pretreated and arsenic intoxicated rats showed significantly less severe changes in tissues from the cerebrum. Degree of congestion, neuronal degeneration was less and neuropil revealed only mild degree of spongiosis (Figure 2e) in comparison to rats given only arsenic. Additionally, meninges did not show any significant alterations. In contrast, quercetin pre‐administration could not ameliorate arsenic neurotoxicity and rats co‐treated with arsenic and quercetin showed more widespread and a higher degree of neuronal degeneration, necrosis, neuronophagia along with spongiosis (Figure 2f) in comparison to the changes seen in rats administered with ZO along with arsenic. However, changes in the latter group of rats were less severe than that in arsenate only treated rats.

**FIGURE 3:**
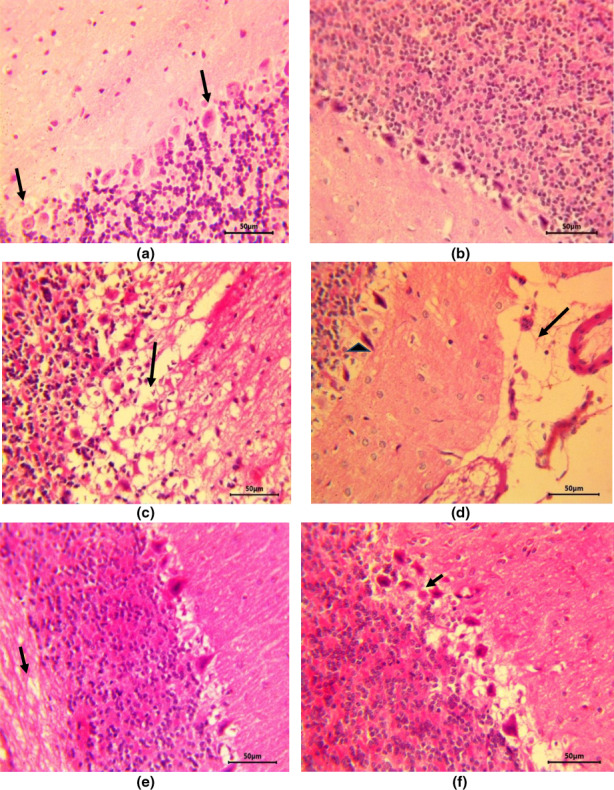
Histology of normal cerebellum with Purkinje cells and granule cell neurons were observed in group I (a) and no pathological alterations were noted in group II (b). Group III indicated severe spongiosis and degeneration of Purkinje cells (c) and meningeal edema (arrow) and degeneration of Purkinje cells (arrowhead) (d). Group IV showed mild spongiosis (arrow) and Purkinje cell degeneration (e) and group V showed spongiosis and degeneration of Purkinje cells (arrow) (f).

In cerebellar sections from control and ZO alone treated rats, all the three layers of cerebellar cortex including Purkinje cell layer were found to be normal (Figure 3a,b) and the white matter did not reveal any abnormalities. Sodium arsenate caused severe pathological alterations including neuronal degeneration in all the cell layers. Purkinje cells were degenerated and neuropil as well as white matter revealed severe spongiosis (Figure 3c) alongside congestion and hemorrhage. Other changes witnessed in this group were gliosis, degeneration and loss of Purkinje cells, and meningeal edema (Figure 3d). In contrast to arsenate treatment, the changes were significantly lessened in group given ZO pretreatment alongside arsenate and included mild neuronal degeneration and mild vacuolation of neuropil (Figure 3e) along with dilation of myelin sheaths in white matter. Quercetin treatment prior to sodium arsenate administration led to the development of moderately severe vacuolation and Purkinje cell degeneration (Figure 3f) along with congestion. Although, quercetin reduced the severity of arsenic‐induced neuronal injury and spongiosis, it was less effective in reducing the arsenic‐induced severe lesions compared with ZO treatment.

## 4 DISCUSSION

Arsenic is a ubiquitous highly toxic metalloid which has attracted attention of the researchers in the past few decades due to its ability to inflict serious health problems in the mammals. Exposure to arsenic causes multi‐systemic toxicity including neurotoxicity in the mammals. Brain is particularly vulnerable to arsenic‐induced deleterious effects because arsenic readily evades blood brain barrier. The GLUT‐1 receptors particularly located in the endothelial cells of blood–brain barrier promote the translocation of inorganic arsenic. The aquaglyceroporins 7 and 9 play a vital role in the uptake of arsenicals into the brain (Liu et al., 2002). Arsenic induces generation of excessive amounts of a variety of ROS/RNS, such as hydrogen peroxide, singlet oxygen, NO, superoxide anion, and hydroxyl radicals primarily through disruption of mitochondrial redox homeostasis and upregulation of NADPH oxidase (Brinkel et al., 2009; Hu et al., 2020; Jomova et al., 2011). Being abundant in polyunsaturated lipids, brain is highly susceptible to arsenic‐mediated oxidative insult. Apart from impairing mitochondrial stability in the nervous tissue, arsenic also recruits pro‐inflammatory cytokines, promotes pro‐apoptotic signaling, and diminishes the protective antioxidant enzyme contents and thereby inflicts severe neuronal damage and directly affects cortex and cerebellum (Medda et al., 2020).

The previous studies suggest that toxic manifestations of arsenic are also due to its direct binding to sulfhydryl (‐SH) groups (Hu et al., 2020). In the present study, arsenic treatment caused a significant reduction in the concentration of ‐SH groups, which may have further reduced the scavenging of arsenic‐generated free radicals. The reduced TAS levels following sodium arsenate administration indicate the suppression of antioxidant defense of the brain tissue. Reduced cellular level of thiols are likely interfered with the activity of GPx along with enhanced ROS‐induced oxidation of brain lipids and proteins which might have been responsible for oxidative injury in brain tissue of rats in the current study. Moreover, reduced GR activity in rat brains after sodium arsenate administration might have compromised the regeneration of reduced form GSH. Arsenic exposure in the present study decreased the activities of SOD and CAT in brain tissue indicating reduced scavenging of these radicals from brain tissue. Increased production and reduced scavenging of ROS/RNS in brain during arsenic intoxication led to oxidative damage to cellular lipids and proteins as indicated by increased production of MDA and AOPP. Arsenic exposure has been reported to increase lipid peroxidation and reduce CAT and GPx in brain along with up regulating heat shock proteins (Zhao et al., 2017).

AE is an enzyme important in catalysis of free radicals and its activity has been reported to get affected by oxidative stress induced by arsenic toxicity (Zargari, 2019). We also noticed a significant decline in AE in rats after sodium arsenate administration and was similar to the reported findings (Flora, 2011). Use of antioxidants like N‐acetyl cysteine for therapeutic management of arsenic poisoning has been advocated to reduce the risk of side effects associated with traditional antidotes of heavy metal toxicities. Extensive research is currently pursued to devise interventions using plant‐derived agents rich in antioxidant properties to negate heavy metal toxicity. Concomitant administration of *Moringa oleifera* in mitigating the effects of arsenic‐induced oxidative stress has been observed as reflected by restored depleted concentrations of GSH, GPx, CAT and diminution of augmented ROS, lipid peroxidation, and arsenic uptake by brain (Gupta et al., 2007).

Pretreatment with ZO extract in our study restored the depleted activities of CAT and SOD and levels of TAS and TTH which may have been responsible for the restoration of GPx and GR activities and diminution of the ROS/RNS‐induced oxidative damage in brain as indicated by reduced MDA and AOPP levels. Natural antioxidants in turmeric have been found effective in combating ROS‐induced arsenic toxicity (Bahrami et al., 2020). The presence of phytochemicals with strong antioxidant properties in ZO extract might be responsible for the remediation of disrupted antioxidant parameters in brain tissue induced by sodium arsenate.

Arsenic‐induced free radical‐mediated lipid and protein oxidation in the brain in our study was also corroborated by histopathological assessment of brain tissue. Both cerebrum and cerebellum from arsenic alone administered rats showed toxigenic insult in the form of neuronal cell damage, perivascular edema, gliosis and spongiosis with congestion, hemorrhage and lymphocytic infiltration in meninges. The ZO pretreatment showed evidence of relatively less oxidative damage of brains as the histopathological alterations such as neuronal degeneration and spongiosis were markedly milder. This implies that antioxidant properties in ZO protected cerebrum and cerebellum considerably from ROS/RNS‐mediated oxidative damage and largely protected architecture of brain during arsenic poisoning. Similar to our results, gliosis and nuclear pyknosis (Peruru et al., 2020) and edema (Noman et al., 2015) have been reported in mice brain during experimental arsenic toxicity. Previous studies (Singh et al., 2020) have reported similar findings such as neuronal degeneration, gliosis, and neuronophagia in rats after subacute exposure to arsenic.

Very high levels of NO in toxicant treated group in our study may be attributed to inflammatory iNOS induction that often competes with O_2_ causing NO‐dependent hypoxia/nitroxia. Nitroxia leads to the generation of excess levels of ROS/RNS which in turn can shut down mitochondrial respiration by irreversible inhibition of electron transport chain (ETC) complexes resulting in depleted levels of ATP and consequent cytotoxic effects. Irreversible inhibition of ETC results in apoptosis and necrosis of brain tissue through energy depletion which may end up in multiple organ failure associated with accelerated and refractory anaerobic metabolism (Levine et al., 2012). Role of iNOS and consequent elevated levels of NO in pathological conditions has been reported in studies (Knott & Bossy‐Wetzel, 2009; Levine et al., 2012; Vallance, 2003). However, increase in levels of NO in group treated with ZO alone may be due to eNOS which is beneficial and responsible for enhanced blood flow to the nervous tissue. Increased NO levels by induction of eNOS by the ZO extract has been reported (Goudarzi et al., 2018; Hasani et al., 2019).

Similar to ZO extract, pretreatment with quercetin also diminished arsenic‐mediated alterations in concentrations of enzymatic antioxidants, markers of protein and lipid peroxidation and histopathological alterations in the brain of rats. However, ZO extract was found to be more potent than quercetin in restoring the levels of SOD, GPx, GSH, and AE which declined after arsenic toxicity. The ZO extract was relatively more efficient in managing the elevated AOPP concentrations and salvaging brain from arsenic‐induced histopathological modifications. In contrast, quercetin was more beneficial in warding off changes in GR, TAS, and MDA levels.

## 5 CONCLUSIONS

Based on our results, it can be concluded that quercetin and ZO extract have neuroprotective potential and significantly offset the effects of arsenic‐induced neurotoxicity. Overall, the ZO extract was more effective than quercetin in lessening the arsenic‐induced neuronal oxidative stress and burden of histopathological lesions in brain. Inclusion of quercetin‐rich foods and ZO extract in human diet may help in combating the arsenic‐induced neurotoxicity in the areas with elevated arsenic levels in food chain and water supply.

## FUNDING INFORMATION

This work was supported by the grant provided by the Indian Council of Medical Research (ICMR), New Delhi, India (Sanction order No. 36/6/2020/TOXI/BMS dated 16/11/2021).

## CONFLICT OF INTEREST STATEMENT

The authors declare that there is no conflict of interest.

## Data Availability

The data that support the findings of this study are available from the corresponding author upon reasonable request.
